# Neuroretinal hypoxic signaling in a new preclinical murine model for proliferative diabetic retinopathy

**DOI:** 10.1038/sigtrans.2016.5

**Published:** 2016-04-22

**Authors:** Katherine J Wert, Vinit B Mahajan, Lijuan Zhang, Yuanqing Yan, Yao Li, Joaquin Tosi, Chun Wei Hsu, Takayuki Nagasaki, Kerstin M Janisch, Maria B Grant, MaryAnn Mahajan, Alexander G Bassuk, Stephen H Tsang

**Affiliations:** 1 Bernard and Shirlee Brown Glaucoma Laboratory and Barbara & Donald Jonas Laboratory of Regenerative Medicine, Columbia University, New York, NY, USA; 2 Edward S. Harkness Eye Institute, Columbia University, New York, NY, USA; 3 Institute of Human Nutrition, Columbia University, New York, NY, USA; 4 Department of Ophthalmology and Visual Sciences, University of Iowa, Iowa City, IA, USA; 5 Omics Laboratory, University of Iowa, Iowa City, IA, USA; 6 Department of Pharmacology and Therapeutics, University of Florida, Gainesville, FL, USA; 7 Eugene and Marilyn Glick Eye Institute, Department of Ophthalmology, Indiana University School of Medicine, Indianapolis, IN, USA; 8 Department of Pediatrics, University of Iowa, Iowa City, IA, USA; 9 New York Presbyterian Hospital/Columbia University Medical Center, New York, NY, USA; 10 Department of Pathology and Cellular Biology, Columbia University, New York, NY, USA

## Abstract

Diabetic retinopathy (DR) affects approximately one-third of diabetic patients and, if left untreated, progresses to proliferative DR (PDR) with associated vitreous hemorrhage, retinal detachment, iris neovascularization, glaucoma and irreversible blindness. In vitreous samples of human patients with PDR, we found elevated levels of hypoxia inducible factor 1 alpha (HIF1α). HIFs are transcription factors that promote hypoxia adaptation and have important functional roles in a wide range of ischemic and inflammatory diseases. To recreate the human PDR phenotype for a preclinical animal model, we generated a mouse with neuroretinal-specific loss of the von Hippel Lindau tumor suppressor protein, a protein that targets HIF1α for ubiquitination. We found that the neuroretinal cells in these mice overexpressed HIF1α and developed severe, irreversible ischemic retinopathy that has features of human PDR. Rapid progression of retinopathy in these mutant mice should facilitate the evaluation of therapeutic agents for ischemic and inflammatory blinding disorders. In addition, this model system can be used to manipulate the modulation of the hypoxia signaling pathways, for the treatment of non-ocular ischemic and inflammatory disorders.

## Introduction

Diabetes is the leading cause of blindness among the working-age population,^1^ with approximately one-third of diabetic patients displaying signs of diabetic retinopathy (DR).^2,3^ Clinically, DR is classified into two progressive stages. The first stage is non-proliferative DR, characterized by intraretinal microvascular abnormalities, microaneurysms and hard exudates. Within 10 years, ~60% of non-proliferative DR patients progress to the second stage, proliferative DR (PDR),^2,4^ during which capillary dropout renders the retina ischemic. If left untreated, patients with PDR can develop retinal neovascularization (angiogenesis), vitreous hemorrhage, intraocular fibrosis, tractional retinal detachment, retinal degeneration, neovascular glaucoma and eventual blindness.^2,5^

Cadavers are currently the only source of human retinal neurons affected by PDR, so a murine model neuroretinal hypoxic signaling in a new preclinical murine model for proliferative diabetic retinopathy would be a powerful tool for testing the progressive clinical phenotype, neovascularization and ischemia. Most available mouse models of DR are inadequate, because within the lifespan of the diabetic animals, they develop only a mild, non-proliferative disease.^6,7^ For example, although the O_2_-induced retinopathy and Kimba/Akimba vascular endothelial growth factor (VEGF) transgenic mouse models have been useful for studying retinopathy of prematurity and vascular leakage, in these mice, neovascularization spontaneously regresses and so limits testing of many therapeutic hypotheses.^2,8–13^ Although the newest murine model for DR (a *Per2* mutant) displays gene expression patterns similar to DR human patients, the mice do not develop the vascular abnormalities characteristic of human PDR.^14^

To develop an appropriate murine model for human PDR, we characterized protein profiles in human vitreous samples, comparing DR patients and control subjects. Our goal was to find a pathway that, when dysregulated, causes retinal hypoxia and leaky neovascularization, key pathologic symptoms of PDR. We determined that hypoxia inducible factors (HIFs) are highly elevated in PDR patient vitreous samples. HIFs are αβ-heterodimeric transcription factors that have a key role in the regulation of tissue metabolism, stress and the adaptation to hypoxic conditions.^15^ HIFs have gained interest in pharmacological studies for their role in a wide range of ischemic and inflammatory diseases, such as renal anemia, inflammatory bowel disease, wound healing and many others. At adequate tissue oxygen levels, HIFs are rapidly degraded via the E3 ubiquitin ligase proteasomal pathway by interaction with the von Hippel Lindau tumor suppressor protein (VHL). Therefore, we created a conditional knockout of VHL in the neuroretina of mice, and found that the photoreceptor cells released elevated levels of HIF1α that led to a severe, progressive, neovascular, PDR phenotype in the mouse, creating a model for the understanding of HIF signaling pathways and the testing of therapeutic agents for the treatment of PDR and other ischemic diseases.

## Materials and methods

### Human cases

The collection of data used in this study was approved by the Institutional Review Board for Human Subjects Research at the University of Iowa, was compliant with the Health Insurance Portability and Accountability Act, and adhered to the tenets of the Declaration of Helsinki. Written informed consent was received from participants before inclusion in the study. Clinical examination and testing were performed as previously described.^16^ Stereoscopic color fundus images and infrared images were obtained using a Topcon TRC 50DX camera (Topcon, Paramus, NJ, USA).

### Enzyme-linked immunosorbent assay

Enzyme-linked immunosorbent assay was performed using the human/mouse total HIF-1 alpha DuoSet IC (DYC1935; R&D Systems, Inc., Minneapolis, MN, USA).

### Mouse lines and husbandry

All animal studies were conducted in accordance with the Association for Research in Vision and Ophthalmology Statement for Use of Animals in Ophthalmic and Vision Research as well as the Policy for the Use of Animals in Neuroscience Research of the Society for Neuroscience, and procedures were approved by the Institutional Animal Care and Use Committee of Columbia University. All mice were housed in the Columbia University Pathogen-free Eye IC and maintained in temperature-controlled rooms with a 12/12-h light/dark cycle. The *Tg(Chx10-EGFP/cre, ALPP)2Clc/J* (called *Chx10-cre* mice for the remainder of this manuscript), *Vhl*^*tm1Jae*^*/Vhl*^*tm1Jae*^ (called *Vhl*^*flox/flox*^ mice for the remainder of this manuscript) and C57BL/6J mice were obtained from the Jackson Laboratory (Bar Harbor, ME, USA). *Tg(Chx10-EGFP/cre, ALPP)2Clc/J* and *Vhl*^*tm1Jae*^*/Vhl*^*tm1Jae*^ mice were crossed for multiple generations to make homozygous *Tg(Chx10-EGFP/cre, ALPP)2Clc/J*×*Vhl*^*tm1Jae*^*/Vhl*^*tm1Jae*^ mice, named *Chx10-cre; Vhl*^*flox/flox*^ mice for the remainder of this manuscript.

### PCR analysis

Genotyping was performed with complementary DNA isolated from either the tails or retinas. The primers, reaction/components and cycling conditions were taken from the Jackson Laboratory website (www.jax.org).

### Quantitative real-time PCR analysis

Retinal extracts from C57BL/6J and *Chx10-cre; Vhl*^*flox/flox*^ transgenic mice were collected at postnatal days (P) 2, 5, 7 and 21. RNA was collect using the RNeasy Mini kit (Qiagen, Germantown, MD, USA). Complementary DNA was made using the SuperScript III First Strand Synthesis SuperMix for qRT-PCR (Invitrogen, Grand Island, NY, USA). Samples were run on a 96-well plate using TaqMan Real-time PCR assays on the Applied Biosystems ABI7500 Real-time PCR machine (Applied Biosystems, Grand Island, NY, USA). Cycling threshold (Ct) values were used to determine the delta Ct and delta–delta Ct values for analysis as previously published.^17,18^

### Immunoblot analysis

Retinal extracts from P10 mice were normalized for protein content (40 μg) and were incubated with the following primary antibodies: anti-von Hippel Lindau N-terminal (1:100; Abcam, Cambridge, MA, USA), anti-HIF-1 alpha (1:1000; Novus Biologicals, Littleton, CO, USA) and rabbit anti-cytoskeletal actin (1:300; Bethyl Laboratories, Inc., Montgomery, TX, USA) at 4 °C overnight. Membranes were probed with goat anti-rabbit IgG-HRP (1:2000; Santa Cruz Biotechnology, Inc., Dallas, TX, USA). All further methods are previously described.^19^

### Frozen sections and whole mounts

Mice were killed according to the established Institutional Animal Care and Use Committee guidelines. Eyes were enucleated, incubated in 2% paraformaldehyde at room temperature for 1 h, and then washed twice with phosphate-buffered saline (PBS; Invitrogen, Carlsbad, CA, USA). For retinal sections, eyes were equilibrated in sucrose, embedded in optimum cutting temperature compound (Sakura Fineteck, Torrance, CA, USA) and frozen. Frozen eyes were sectioned at 10 μm. For retinal whole mounts, the optic nerve, cornea and lens were removed and the entire eyecup was flattened by making four radial cuts that extended out from the optic nerve.

### Immunostaining

Sections were permeabilized with 0.1% Triton-X100 (Fisher Scientific, Fair Lawn, NJ, USA) in PBS (PBS-T) and blocked in 5% goat serum. Sections were stained with one of the following primary antibodies: CD31 (1:20; BD Biosciences, San Jose, CA, USA), VEGFA (1:1000; Abcam), HIF1α (1:500; Novus Biologicals) and VHL (1:100; Santa Cruz Biotechnology). Secondary antibodies were Cy3-conjugated donkey anti-Rat (Jackson ImmunoResearch Laboratories Inc., Bar Harbor, ME, USA), Alexa Fluor 488 donkey anti-rat (Invitrogen), Alexa Fluor 488 and Alexa Fluor 555 goat anti-rabbit (Invitrogen), and Alexa Fluor 555 goat anti-mouse (Invitrogen) at 1:500. Sections were imaged using a Leica DM 5000B microscope at ×2.5 and ×40 magnification (Leica Microsystems Inc., Wetzlar, Germany).

### Electroretinograms

Mice were dark-adapted overnight, manipulations were conducted under dim red light illumination and recordings were made using Espion ERG Diagnosys equipment (Diagnosys LLL, Littleton, MA, USA). Adult C57BL/6J control mice were tested at the beginning of each session to ensure equal readouts from the electrodes for both eyes before testing the transgenic experimental mice. Both eyes were recorded simultaneously. A total of 40–60 responses were averaged for each trial. All further detail on the electroretinogram (ERG) method has been described previously.^19,20^ Unpaired *t*-tests between C57BL6/J controls and transgenic mice were used to determine statistical significance for the *a*- and *b*-wave maximal amplitudes at 1 and 2 months of age.

### Perfusions

Confocal images were collected from mice that had been intraperitoneally injected with a lethal dose of anesthesia (1 ml ketamine 100 mg ml^−1^ (Ketaset III, Fort Dodge, IA, USA) and 0.1 ml xylazine 100 mg ml^−1^ (Lloyd Laboratories, Shenandoah, IA, USA) in 8.9 ml PBS). Incisions opened the chest cavity and exposed the heart. A vacutainer blood collection needle was inserted into the left ventricle of the heart, and the right atrium was clipped with Vannas scissors. Saline (2.5 cc) was delivered through the blood stream via the heart, followed by fluorescein or rhodamine dextran dye to label the blood vasculature. Eyes were enucleated and retinal whole mounts were then processed as above without further immunostaining.

### Histology

Mice were killed according to institutional guidelines. Each eye was rapidly removed, punctured at the 12 o’clock position along the limbus and placed in a separate solution of 2% paraformaldehyde, 2.5% glutaraldehyde, 0.1 M sodium phosphate buffer in PBS (Electron Microscopy Sciences, Hatfield, PA, USA) for 1–2 days. After fixation, the eyes were washed with saline and the 12 o’clock limbal puncture was used to orient the right and left eyes (which were kept separate) so that the posterior segment containing the retina could be sectioned along the vertical meridian. A rectangular piece containing the entire retina from superior to inferior ora serrata, including the optic nerve, was prepared for postfixing via osmic acid, dehydration and upon embedding. A corner was cut from the superior ora to mark the upper retinal half of the segment for orientation. Sectioning proceeded along the long axis of the segment so that each section contained upper and lower retina as well as posterior pole. Semi-serial sections were stained with either hematoxylin and eosin or toluidine blue, mounted and examined by light microscopy.

### Fundoscopy and iris angiography

*In vivo* microscopy and digital imaging were performed as described previously,^21^ using an M2Bio fluorescence stereo microscope Stemi SV1 (Carl Zeiss, Oberkochen, Germany). For *in vivo* observation, mice were anesthetized with 3% isoflurane in oxygen. The eye was lightly proptosed and Viscotears (Novartis, Basel, Switzerland) was applied to the cornea after which a circular 7.5-mm sapphire window (Edmund Optics, Barrington, NJ, USA) was placed over the cornea. Fundus images were collected with a digital camera through a ×1.6 objective and a zoom at ×2 or ×4 magnification (Coolsnap ES, Roper, TX, USA). Image resolution was ~1.0 and 2.0 μm per pixel with the ×4 and ×2 magnification, respectively. Fluorescent images were acquired under identical conditions. The intensity of excitation light at 430 nm was monitored with a power meter (Optical Power Meter Model 840, Newport Corp., Irvine, CA, USA) to ensure reproducibility. Serial fluorescein angiography was used with argon blue illumination and a fluorescein barrier filter. One bolus containing 0.2 ml fluorescein (100 mg ml^−1^) was injected into the tail vein.

### Retinal angiography and autofluorescence imaging

Angiography and AF imaging were obtained with the Spectralis optical coherence tomography scanning laser confocal ophthalmoscope (OCT-SLO Spectralis 2; Heidelberg Engineering, Heidelberg, Germany) as previously described.^22,23^ In brief, pupils were dilated using topical 2.5% phenylephrine hydrochloride and 1% tropicamide (Akorn Inc., Lakeforest, IL). Mice were anesthetized by intraperitoneal injection of ketamine/xylazine as described above. During the procedure, body temperature was maintained at 37 °C using a heating pad. A measure of 0.05 ml of 10% AK-Fluor (100 mg ml^−1^, Akorn Inc.) was injected intraperitoneally. Imaging was obtained at 488-nm absorption and 495-nm emission using a 55° lens. Images of the central retina were taken, with the optic nerve located in the center of the image.

### Statistics

All statistics used the two-tailed unpaired *t*-test and error bars display the mean value±s.d.’s. *P*<0.05 was considered to be statistically significant.

## Results

### Vitreous samples from DR patients contain elevated levels of HIF1α

**Figure 1:**
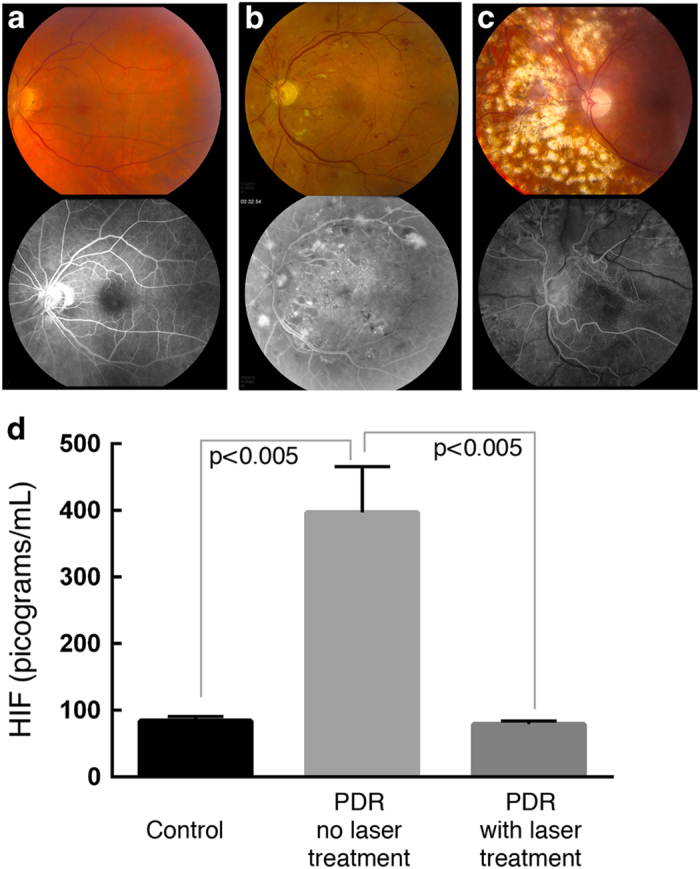
Increased HIF1α protein levels in human patients with untreated proliferative diabetic retinopathy (PDR). Fundus (top) and fluorescein angiographic images (bottom) of the retinae of patients without PDR (**a**), PDR patients who were untreated (**b**) and patients with PDR who underwent prior panretinal photocoagulation (PRP) laser treatment (**c**). Enzyme-linked immunosorbent assays on vitreous samples taken from each patient group determined that HIF1α protein levels were similar in the vitreous of both control patients and treated PDR patients, but significantly elevated (*P*<0.005) in PDR patients who were not treated at the time of analysis (**d**). *N*=4 patients without PDR, 3 patients with untreated PDR and 3 patients with PDR who underwent PRP laser treatment. Error bars represent s.d.’s.

Human DR patient retinal tissue has previously been shown to upregulate both erythropoietin (EPO) and VEGF, suggesting a potential role of HIF1α in the progression of DR.^24^ Furthermore, HIFs have recently been linked to a wide range of diseases, such as inflammatory diseases, ischemic disorders, metabolic syndrome and type 2 diabetes.^15,25^ To determine whether HIF1α was active in human PDR, we collected vitreous from patients with DR, as it has been shown that the retina releases proteins into the vitreous.^26,27^ During vitrectomy surgery, we collected samples from patients with active PDR (three patients) and two control sets of patients: those without DR (four patients) and patients in which prior panretinal laser photocoagulation (PRP) was used to ablate the ischemic retina and inhibit neovascularization (three patients; Figure 1).^22^ Untreated PDR patients showed early signs of neovascularization, exudates and hemorrhages on both fundus (top) and angiography images (bottom; Figure 1b). For PRP-treated DR, patients showed signs of laser scarring that was visible in after-surgery images of the fundus (top) and angiography images (bottom; Figure 1c), but no neovascularization.

Enzyme-linked immunosorbent assays revealed low levels of HIF1α in the vitreous from control samples (Figure 1d). In PDR patients who had not received any PRP laser therapy, there was significantly elevated levels of HIF1α (*P*<0.005). In PDR patients who had received PRP laser therapy with successful regression of neovascularization, the levels returned to baseline. These results were intriguing, as *HIF1α* is a ubiquitous transcription factor that drives expression of genes such as *EPO*, *VEGF*, *CXCR12* and nitric oxide synthase (all of which cause neovascularization) and regulates adaptive responses to hypoxia (that is, glycolysis, erythropoiesis, angiogenesis and vascular remodeling).^23,28,29^

### Establishment of a preclinical model of PDR

As there was elevated HIF1α expression in human PDR eyes, overexpression of HIF1α specifically in the mouse neuroretina might offer a preclinical, DR model. To elevate retinal HIF1α, we chose its natural upstream regulator, VHL. Under normoxic conditions, VHL marks HIF1α for ubiquitin-mediated degradation.^30,31^ We ablated VHL specifically in retinal neurons (global VHL expression causes embryonic lethality)^31,32^ and expected this would constitutively elevate HIF1α levels in the retina.

**Figure 2:**
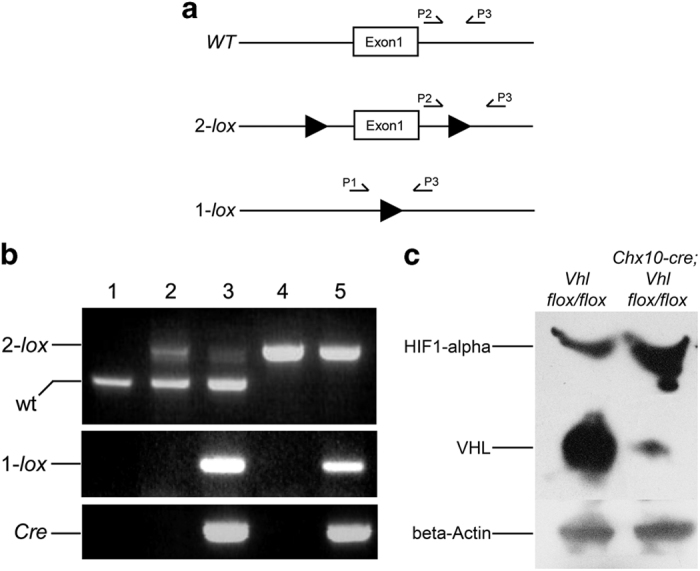
Increased HIF1α but decreased VHL levels in the *Chx10-cre; Vhl*^*flox/flox*^ mutant mice. A schematic of the *Vhl* gene, displaying exon 1 and the *loxP* sites of the *Vhl*^*flox/flox*^ mice shows the location of the primers used to detect *cre*-mediated recombination after cross-breeding with *Chx10-cre* mice (**a**). Primers P2 and P3 detect the *loxP* sites in retinal DNA samples. Primers P1 and P3 detect *cre*-mediated recombination. PCR analysis of retinal samples after cross-breeding of the *Vhl*^*flox/flox*^ mice with the *Chx10-cre* mice (**b**). A 290-bp band is visible for wild-type alleles (sample 1), whereas a 460-bp band is visible for the 2-lox allele (samples 4 and 5), and a double band at both sites is detected in heterozygous allele mice (samples 2 and 3), which harbor one wild-type allele and one with 2-lox sites. *Cre* expression was found in samples 3 and 5, both of which displayed *cre*-mediated recombination to produce a 1-lox mutant allele (signal at 290 bp). Immunoblot analysis of HIF1-alpha and VHL protein levels in *Vhl*^*flox/flox*^ *cre-*negative control mice (lane 1) and *Chx10-cre; Vhl*^*flox/flox*^ mutant mice (lane 2) at postnatal day 10 (**c**). β-Actin was used as a loading control.

Mice deficient for retinal VHL (VHL knockouts) were derived from *Vhl*^*flox/flox*^ mice (Jackson Laboratory) that harbor *Vhl* alleles flanked by *loxP* sites. These mice were crossed with *Chx10-cre* mice, which express *cre* specifically in the fetal neuroblasts, photoreceptors and adult bipolar cells of the neuroretina. PCR analysis verified retinal *Vhl* knockout in the resultant offspring (Figures 2a and b).

### HIF1α protein levels are elevated in the *Chx10-cre; Vhl*^*flox/flox*^ murine model

**Figure 3:**
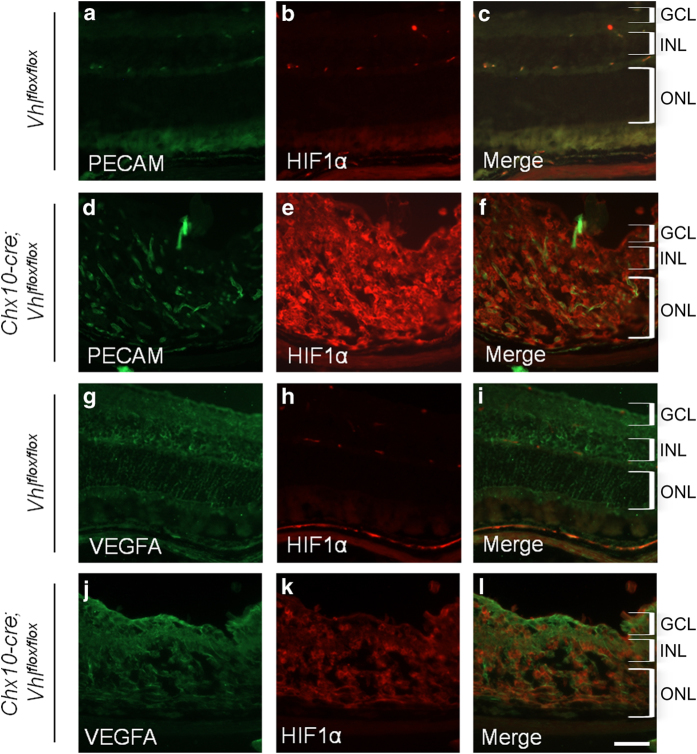
*Cre*-mediated recombination causes increased HIF1α. Protein levels were analyzed by immunostaining frozen retinal sections for *Vhl*^*flox/flox*^ *cre-*negative mice and *Chx10-cre; Vhl*^*flox/flox*^ mutant mice at 1 month of age. Both PECAM (green; **a**) and HIF1α (red; **b**) protein levels were present in *Vhl*^*flox/flox*^ *cre-*negative mice, but PECAM (**d**) and HIF1α (**e**) protein levels were only elevated after *cre*-mediated excision of the *Vhl* gene. Merged images for the *Vhl*^*flox/flox*^ *cre*-negative mice (**c**) and *Chx10-cre; Vhl*^*flox/flox*^ mutant mice (**f**) show combined PECAM (green) and HIF1α (red) protein levels in the retina. Vascular endothelial growth factor (VEGF; green) was similar between the *Vhl*^*flox/flox*^ *cre-*negative mice (**g**) and *Chx10-cre; Vhl*^*flox/flox*^ mutant mice (**j**), whereas HIF1α (red) was increased as expected in the mutant mice (**k**) compared with the *cre*-negative mice (**h**). Merged images for the *cre*-negative mice (**i**) and mutant mice (**l**) show VEGFA and HIF1α overlayed on the same figure. Both the sections for PECAM and HIF1α, and those for VEGFA and HIF1α were taken at the same exposure. GCL, ganglion cell layer; INL, inner nuclear layer; ONL, outer nuclear layer. Scale bar, 600 μm.

To assess whether the neuroretina knockout of the *Vhl* gene caused protein changes in mice that phenocopied human DR patients, protein levels in the retina were analyzed in *Vhl*^*flox/flox*^ *cre*-negative mice and *Chx10-cre; Vhl*^*flox/flox*^ mutant mice at P10 (Figure 2c). Compared with the control, VHL was significantly reduced in the mutant mouse, although a residual level of VHL was still detectable (Figure 2c). More importantly, as in human DR patients (Figure 1), mutant mice greatly upregulated HIF1α expression in the retina (Figures 2c and 3a–f), supporting the notion that the VHL knockout mouse offers a preclinical model for DR and ischemic retinopathies. Platelet endothelial cell adhesion molecule (PECAM), marking the blood vasculature, is found infiltrating the retinal cell layers, which are highly disorganized, in the transgenic mice in comparison with the control mice (Figures 3a–f). Despite these changes, VEGF protein, a downstream target of HIF1α transcriptional activation, remained unchanged in the mutant relative to control mice (Figures 3g–l).

### Photoreceptor elevation of HIF1α leads to upregulation of the downstream transcriptional targets, EPO and *Vegf*, in the *Chx10-cre; Vhl*^*flox/flox*^ mutant mouse retinas after 1 week of age

**Figure 4:**
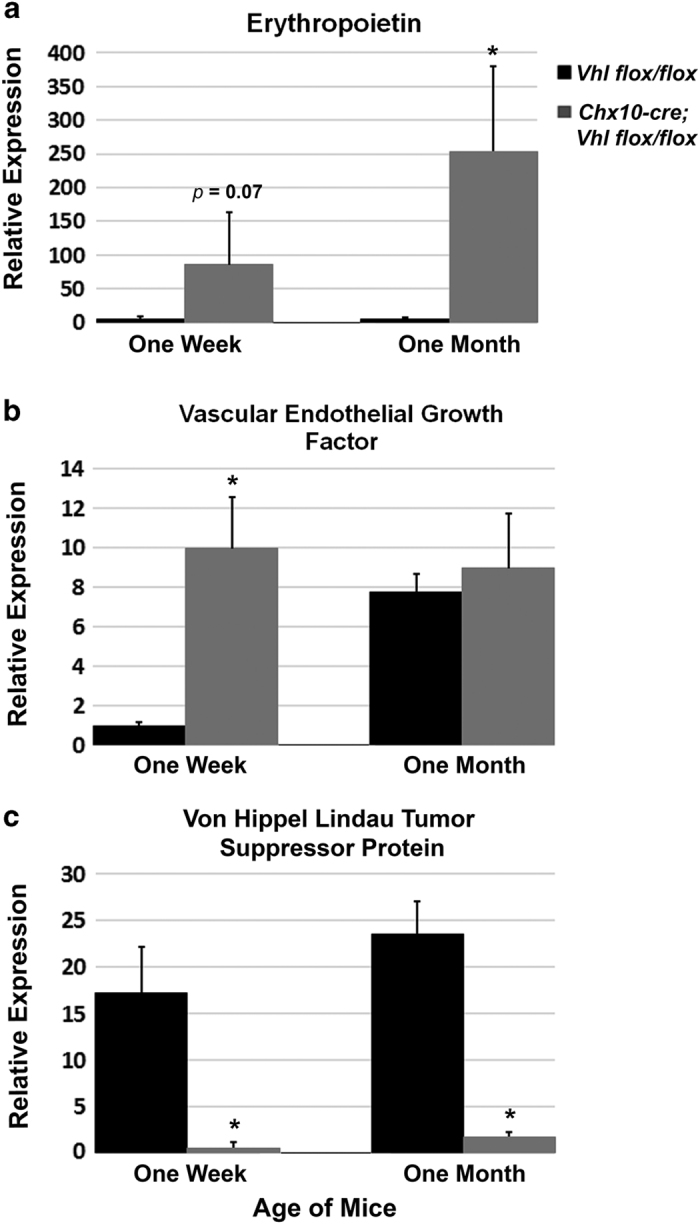
Upregulation of erythropoietin and vascular endothelial growth factor in the PDR murine model. Quantitative PCR of erythropoietin (EPO) showed an upregulation of EPO in the *Chx10-cre; Vhl*^*flox/flox*^ mutant mouse retinas compared with *Vhl*^*flox/flox*^ control mouse retinas at 1 week of age, during the onset of neovascularization and the PDR disease phenotype (**a**). The upregulation of EPO continued through 1 month of age, when hemorrhaging and retinal detachment can be found in this mouse model (**a**). Vascular endothelial growth factor (*Vegf*) was highly upregulated in the *Chx10-cre; Vhl*^*flox/flox*^ mouse retinas at the onset of disease (1 week of age), but became comparable to the control *Vhl*^*flox/flox*^ mouse retinas by 1 month of age (**b**). von Hippel Lindau tumor suppressor protein (*Vhl*) was reduced in the mutant *Chx10-cre; Vhl*^*flox/flox*^ mouse retinas compared with the control *Vhl*^*flox/flox*^ mouse retinas at all time points analyzed as would be expected with the expression of *cre* (**c**). *N*⩾4 for each group at each time point. Error bars show s.d.’s. *P<0.01.

As VEGF is a secreted protein and the changes in VEGF protein levels may not be detectable within the retina, we performed quantitative PCR to test for EPO and *Vegf* messenger RNA expression (Figure 4). Retinas were extracted from mice during early and late stages of the disease phenotype. At 1 week of age (P7), when neovascularization has become visible in the mutant mouse eyes, EPO expression was beginning to be upregulated in the mutant mouse retinas in comparison with *cre*-negative control retinas (Figure 4a). At 1 week of age, there is also a significant upregulation of *Vegf* in the retinas of the mutant mice in comparison with the *cre*-negative control retinas (Figure 4b). At 1 month of age (P28), when the PDR phenotype is severe and some retinal detachment can be found in the mutant murine model, EPO remained highly upregulated in the *Chx10-cre; Vhl*^*flox/flox*^ mutant mouse retinas in comparison with a control *Vhl*^*flox/flox*^ *cre*-negative mouse retina; however, *Vegf* levels were comparable between the control and mutant mice, verifying the protein staining results (Figures 4a and b). As expected, *Vhl* messenger RNA levels were reduced in the *Chx10-cre; Vhl*^*flox/flox*^ mutant mouse retinas compared with the *Vhl*^*flox/flox*^ *cre*-negative mouse retinas (Figure 4c).

### The *Chx10-cre; Vhl*^*flox/flox*^ murine model exhibits anterior segment ischemia, vitreous hemorrhage and tractional retinal detachment similar to human patients with late-stage PDR

**Figure 5:**
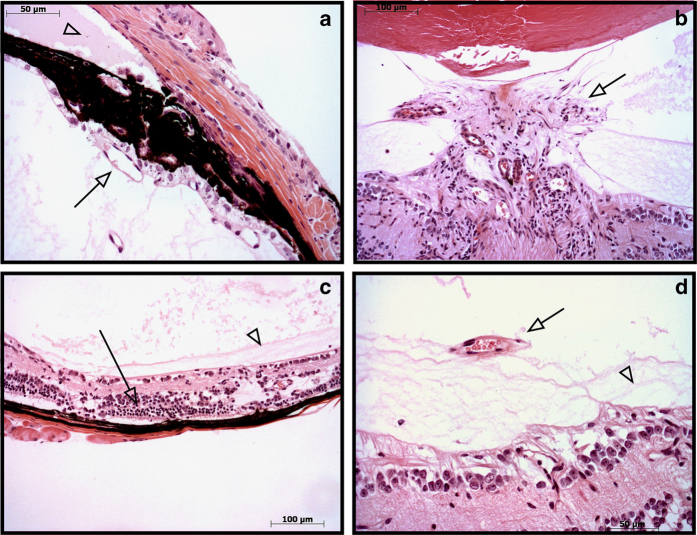
Neovascularization and retinal degeneration in the PDR murine model. Hematoxylin and eosin (H&E)-stained pupil optic nerve sections from eight *Chx10-cre; Vhl*^*flox/flox*^ mice. At 1 month of age, the mice displayed signs of iris neovascularization (arrow) and protein extravasation in the anterior chamber (arrowhead), a sign of vascular leakage (**a**). There was optic disc neovascularization (arrow) and fibrosis in the vitreous (**b**), retinal photoreceptor degeneration (arrow) and vitreoretinal membranes (arrowhead) (**c**). Tractional vitreoretinal membranes (arrowhead) and vitreous neovascularization (arrow) were observed (**d**). *N*=12 eyes. All mice displayed neovascular and retinal degenerative phenotypes.

Within the retina of human DR patients, ischemia, capillary dropout and abnormal angiogenesis cause neovascularization and hemorrhaging. To test whether the *Chx10-cre; Vhl*^*flox/flox*^ mutant mice developed similar abnormalities, histological sections were examined from *Chx10-cre; Vhl*^*flox/flox*^ mice at 1 month of age (Figure 5). Similar to human DR patients, these mice showed neovascularization throughout the eye (Figures 5a, b and d), retinal degeneration with disorganization of the retinal cell layers (Figure 5c), and retinal membranes and traction (Figures 5c and d). Each of these histological findings suggests that these mice exhibit key features of PDR and model advanced stages of the human disease.

### The *Chx10-cre; Vhl*^*flox/flox*^ murine model exhibits a loss of visual function

**Figure 6:**
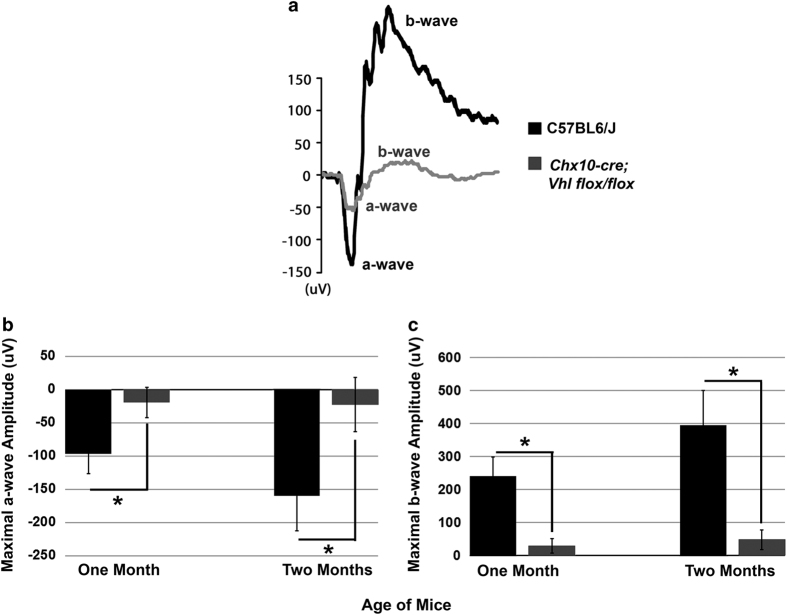
Loss of visual function in the PDR murine model. A representative electroretinogram (ERG) trace at 1 month of age for maximum scotopic rod–cone mixed visual function displayed a severely attenuated visual response in the *Chx10-cre; Vhl*^*flox/flox*^ mutant mice (gray) compared with C57BL/6J wild-type control mice (black; **a**). Quantification of the ERG maximum scotopic *a*-wave amplitude (labeled in **a**) for a cohort of mice displayed a significant loss of *a*-wave amplitude in the *Chx10-cre; Vhl*^*flox/flox*^ mice compared with C57BL6/J controls at both 1 month of age (*P*<0.0001) and 2 months of age (*P*<0.0011; **b**). Similarly, there was a significant loss of the *b*-wave visual function (labeled in **a**) for the *Chx10-cre; Vhl*^*flox/flox*^ mice at both 1 month of age (*P*<0.0001) and 2 months of age (*P*<0.0001; **c**). *N*>5 mice per group for each time point analyzed. Error bars show s.d.’s.

Histological analysis of the *Chx10-cre; Vhl*^*flox/flox*^ mutant mice at 1 month of age displayed abnormal retinal organization (Figure 5). Therefore, we examined visual function using ERGs at both 1 and 2 months of age (Figure 6). Under maximum rod–cone scotopic ERG settings at 1 month of age, the mutant mouse (gray) had a low *b*:*a* wave ratio in comparison with that of the control mouse (black; Figure 6a). A cohort of mice (*n*>5) was then analyzed by ERG at both 1 and 2 months of age. The *a*-wave of the maximum rod–cone scotopic ERG response was significantly reduced at 1 month of age (−96.3±30.2 μV in the control mice and −19.0±22.7 μV in the mutant mice; *P*<0.0001) and the *a*-wave amplitude remained significantly reduced at 2 months of age (−159.4±52.8 μV in the control mice and −22.3±40.5 μV; *P*=0.0011; Figure 6b). In addition, the *b*-wave amplitude of the maximum rod–cone scotopic ERG response was also significantly reduced at both 1 month of age (239.8±59.1 μV in the control mice and 28.1±21.8 μV in the mutant mice; *P*<0.0001) and 2 months of age (394.6±105.2 μV in the control mice and 47.5±29.5 μV in the mutant mice; *P*<0.0001; Figure 6c). Overall, visual function was attenuated in the *Chx10-cre; Vhl*^*flox/flox*^ mutant mice in comparison with wild-type controls, likely due to the retinal ischemia in these mice.

### The *Chx10-cre; Vhl*^*flox/flox*^ murine model exhibits vasculature defects found in human PDR patients

**Figure 7:**
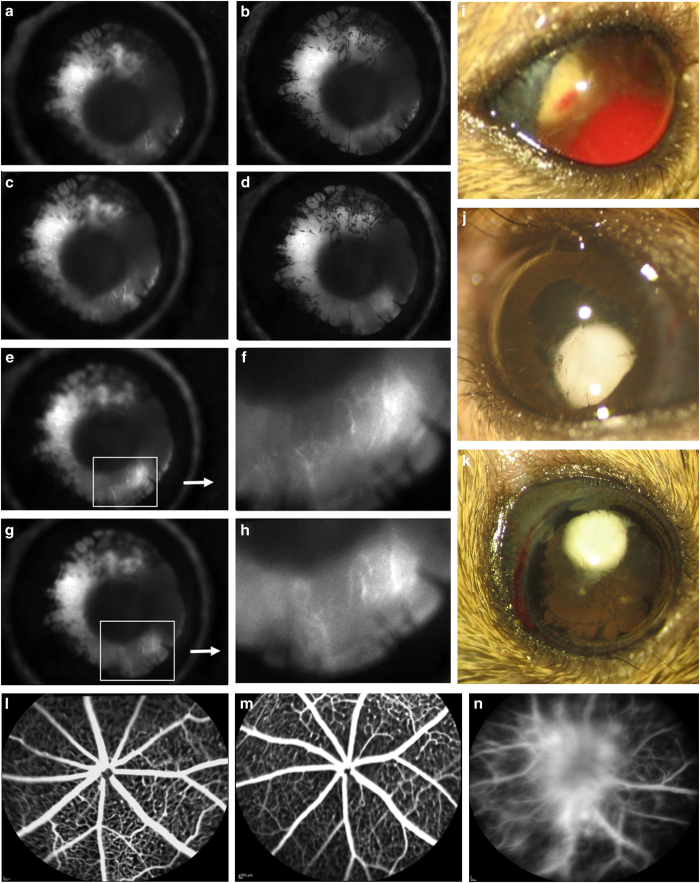
Neovascularization and leakage in the *Chx10-cre; Vhl*^*flox/flox*^ mutant mouse mimics the human proliferative DR (PDR) phenotype. Time-lapse images from iris angiography at postnatal day 10 (P10) reveals anterior segment ischemia, rubeosis and iris synechaie (**a**–**e**, **g**). Enhanced images of fluorescein leakage in the iris during time-lapse angiography (**f**, **h**). *t*=10 s post-fluorescein dye injection. Fundus photographs of mutant mice at 3 months of age, showing visible vitreous hemorrhage (**i**), cataract (**j**) and signs of anterior segment ischemia (**k**). Fluorescein angiography in mice older than 1 month of age, for a wild-type C57BL/6J mouse (**l**) and a *Chx10-cre; Vhl*^*flox/+*^ heterozygote (**m**), uncovered no fluorescein leakage or detectable abnormalities of the retinal vasculature. In contrast, at P19, angiography of the *Chx10-cre; Vhl*^*flox/flox*^ mutant mouse detected leaking fluorescein dye and capillary dropout, characteristic of PDR in humans (**n**). *N*⩾4 mice, phenotype was visible in all mice examined.

In addition, we evaluated the irises of the *Chx10-cre; Vhl*^*flox/flox*^ mutant mice at P10. Angiography revealed that they indeed developed anterior segment ischemia (Figures 7a–d), and in images of time-lapse angiograms (Figures 7e–h), magnification revealed leaking fluorescein dye indicative of iris rubeosis (Figures 7f and h). In addition, the anterior surface of their irises developed a fibrovascular membrane (Figures 7a–d). These findings are consistent with the early development of late-stage PDR in the *Chx10-cre; Vhl*^*flox/flox*^ mutant mouse model.

The neovascularization on the surface of the iris and nearby structures in the *Chx10-cre; Vhl*^*flox/flox*^ mice can elevate intraocular pressure and further damage the optic nerve, as with human neovascular glaucoma. Accordingly, fundus photographs of the mutant mice revealed phenotypes associated with neovascular glaucoma, retinal ischemia and PDR at 3 months of age (Figures 7i–k). Vitreous hemorrhage was clearly visible in ~60% of the mutant mice (Figure 7i), as was cataract formation (Figure 7j) anterior synechia (Figure 7k) and neovascular glaucoma (Figures 7j and k). In addition, retinal vessels in the mutant mice leaked fluorescein dye and demonstrated capillary dropout, suggesting that their condition is similar to human PDR (Figure 7n). In contrast, for both the control C57BL/6J mouse (Figure 7l) and a *Chx10-cre; Vhl*^*flox/+*^ heterozygous mouse (Figure 7m), retinal angiograms showed normal retinal vasculature and no detectable phenotype.

**Figure 8:**
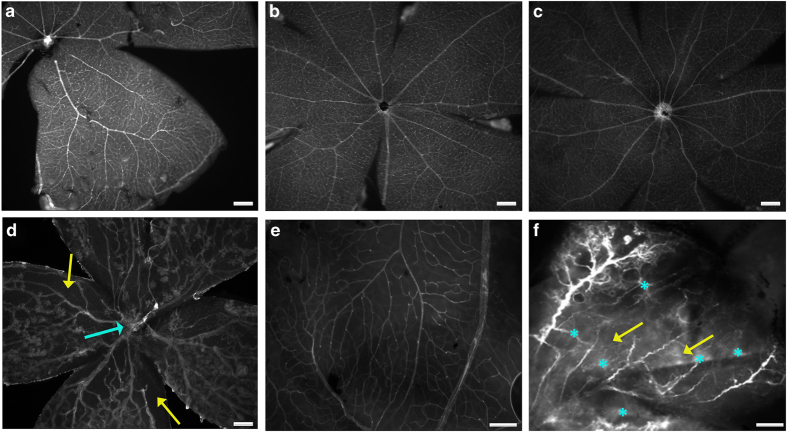
Retinal whole-mount analysis shows *Chx10-cre; Vhl*^*flox/flox*^ mutant mice develop a PDR phenotype. Retinal whole mounts were analyzed at >3 months of age for a C57BL/6J control mouse (**a**), a *Vhl*^*flox/flox*^ mouse model without *cre* expression (**b**) and a *Chx10-cre; Vhl*^*flox/+*^ heterozygous mouse model (**c**). PECAM (CD31) staining revealed that the three controls had developed a normal capillary bed. In contrast, by postnatal day 8 (P8), the homozygous *Chx10-cre; Vhl*^*flox/flox*^ mutant mice had developed severe retinal blood vessel abnormalities (**d**). Rhodamine dextran perfusions displayed normal capillaries at 2 months of age in *Chx10-cre; Vhl*^*flox/+*^ heterozygous mice (**e**), whereas capillary dropout and dye leakage denoting neovascularization is visible by P5 in a homozygous mutant (**f**). Blue asterisk, neovascularization; yellow arrow, capillary dropout; blue arrow, neovascularization of the optic disc. *N*⩾5 mice, with all mice showing represented retinal phenotypes. Scale bar, 200 μm.

To confirm the angiography results, retinas were flat mounted and stained with PECAM (CD31), a vascular endothelial cell marker. In addition, because mutant mouse retinas were susceptible to damage during the staining procedure before the eyes were isolated for analysis, mice were perfused with a rhodamine dextran dye to delineate the retinal vessels (Figure 8). Retinal vasculature of C57BL/6J control mice (Figure 8a), the *Vhl*^*flox/flox*^ mouse model without *cre* expression (Figure 8b) and *Chx10-cre; Vhl*^*flox/+*^ heterozygous mice (Figure 8c) all showed normal retinal blood vessels at >3 months of age. In contrast, by P8, the *Chx10-cre; Vhl*^*flox/flox*^ mutant mice had developed severe retinal blood vessel abnormalities (Figure 8d). In homozygous mutant mice, the primary vascular plexus developed normally until P8; from P8 on, their retinas were affected by capillary dropout, as indicated by non-perfused hypo-fluorescent patches (Figure 8d; yellow arrows). In addition, neovascularization was observed on the optic disc and a neovascular network surrounded the optic nerve (neovascularization of disc; Figure 8d; blue arrow), forming a membrane made of new, fragile blood vessels. In human PDR patients, a similar, fragile neovascular network develops and hemorrhages into the vitreous. Neovascular membranes are responsible for the tractional retinal detachments in humans with PDR.

In contrast to the oxygen-induced retinopathy mouse models used for ischemic retinopathies, there was no revascularization of the retina in our mutant mouse. Indeed, the retinal vasculature abnormalities persisted through at least P150 (data not shown). Closer examination of the retinal capillaries showed that compared with 2-month-old heterozygous *Chx10-cre; Vhl*^*flox/+*^ mice (Figure 8e), the homozygous mutant *Chx10-cre; Vhl*^*flox/flox*^ mice were already losing capillaries at P5 (Figure 8f; yellow arrows) and the perfusion dye leaked from the sites of abnormal and hemorrhaging blood vessels that extended to the peripheral retina (Figure 8f; blue asterisks).

## Discussion

The late-stage clinical phenotype of DR, in particular retinal edema and neovascularization, leads to the adverse effects on vision.^33^ However, animal models for DR are mainly limited to mild retinal disease in comparison with human DR patients. Here we examined human PDR patients with and without treatment and determined that elevated HIF1α was found in patients with PDR. We then developed an improved preclinical animal model for human PDR. Elevated HIF1α from the photoreceptor cells allows for irreversible vascular abnormalities in mice deficient in VHL in the neural retina. In our model, the phenotype begins and progresses similar to the clinical presentation of human PDR during the development and growth of the mouse. This new model provides greater clinical relevance when testing therapeutic treatments for PDR and will be useful for developing novel therapies for other ischemic retinal neovascular diseases, including branch retinal vein occlusions, sickle cell retinopathy and inherited neovascular inflammatory vitreoretinopathy.^17^

Recently, murine models were developed that inactivate VHL within the eye, which may lead to elevation of HIF1α as we have found in our murine model system.^34–37^ Although global knockout of VHL causes embryonic lethality, these conditional knockout models targeted specific cell types of the eye and were viable. An astrocyte-specific knockout of VHL displayed accelerated primary hyaloidal vascular regression and massive secondary outgrowth of hyaloid vessels.^34^ A retinal pigment epithelial-specific knockout of VHL developed abnormal retinal pigment epithelials, irises, ocular growth and vasculature in the anterior chamber.^35^ VHL knockout in the distal retina and iris (via a *Pax6 alpha::cre* driver) caused abnormal vasculature and retinal cell death.^36^ VEGF, VHL and HIF1α conditional deletions in the horizontal and amacrine cells reduced blood vessel branching points and lead to a loss of visual function and photoreceptor cells.^37^ None of these models, however, developed phenotypes that mimic advanced human PDR nor did their vascular phenotype progress from non-PDR to PDR; nevertheless, each displayed abnormal retinal vasculature consistent with the idea that the VHL/HIF1 pathway has a role in DR development and inflammatory/ischemic diseases. These findings support the concept that photoreceptor ischemia is the driving force behind PDR, and specific metabolic modulation of photoreceptors may be a therapeutic strategy for the treatment of DR.

Recent studies have concluded that photoreceptors have a role in the progression of DR before retinal neovascularization, but the exact role of photoreceptors and neurodegeneration are still unknown.^38^ Understanding the pathogenesis of retinal neovascularization in DR patients, including neuronal dysfunction that leads to neurodegeneration and vision loss, is highly important for the prevention of vision loss in patients with diabetes. In our model, *Vhl* was absent and *Hif1α* was elevated in the inner retina during early development. Using various *in vivo* and other experimental methods, such as angiography and histological analysis, we determined that the *Chx10-cre; Vhl*^*flox/flox*^ mutants develop retinal disease in a similar sequence as humans that develop retinal ischemia and PDR (Figures 2, 3, 4, 5, 6, 7, 8). Elevation of EPO, VEGF and vitreous hemorrhage began on approximately P4; and by 3 weeks of age, these mice typically progressed to tractional retinal detachment. This was the direct consequence of severe capillary dropout, the formation of ischemic retinal areas, and fibrovascular membranes, all of which are promoted by HIF1α activity. This preclinical model of PDR can be used to investigate the mechanisms underlying the severe disease phenotypes as it is an early-onset model system with no reversal of the phenotype. For instance, the mechanisms of cell death in DR are still mostly unknown, with some studies showing apoptosis, whereas others suggest various different pathways.^39–42^ In addition, as retinopathy in our new mutant mice progresses in a matter of weeks, these mice offer an excellent model for rapid testing of therapeutic agents for this group of incapacitating disorders and different time points of disease progression could be specifically targeted.^6,43^

In summary, we developed a novel murine model of neovascularization, hemorrhage and retinal ischemia characteristic of human patients with PDR. This mouse model can be used to dissect the mechanisms leading to PDR, and can be used to develop treatments for PDR, as it is the first murine model that has a neovascular and ischemic retinal phenotype that does not reverse over time. Furthermore, our newly developed murine model should contribute to knowledge and discovery of similar retinal neovascular diseases, including the retinopathy of prematurity, age-related macular degeneration and tumorigenesis, as well as the study of normal retinal vasculogenesis and the understanding of the HIF signaling pathways to treat a wide range of inflammatory and ischemic diseases.
